# Supplementary material for: Machine learning plastic deformation of crystals
Source: Nat Commun. 2018 Dec 13;9:5307. doi: 10.1038/s41467-018-07737-2 (PMC6294252; doi:10.1038/s41467-018-07737-2)
Supplement: Supplementary file 1 — Supplementary Information [file 41467_2018_7737_MOESM1_ESM.pdf]

Supplementary Information

Machine learning plastic deformation of crystals

Salmenjoki et al.

Supplementary Note 1: **Machine learning models.** To fit the stress response of the dislocation systems to the used configuration descriptors (for a full list, see Supplementary Table 2), we applied both artificial neural networks (ANN) and support vector machines (SVM). Again for a thorough background of the methods, the reader is referred to e.g. [1] while merely technical details of the implementation are described here.

The ANN was implemented with the TensorFlow library [2] and it is illustrated in Supplementary Figure 1. The used network consisted of three fully connected hidden layers with 20 neurons each using the rectifier as activation function. The training procedure was implemented with the stochastic gradient descent algorithm and it used the early-stopping criterion, i.e., the data set was divided to training, test and validation sets and the validation set measured the score during training to stop the training before overfitting to the training data. The test set was then used to evaluate the final performance of the fit. We conducted the training with multiple random seeds to find the combination of learning rate and initial weights for the best fit and, although one training run of the network was not too slow (less than an hour), finding at least somewhat desirable parameters required tens of runs.

For the SVM, we used the ready-made implementation of Scikit-learn [3]. In short, the idea of SVMs is to find support vectors that represent the data the best way possible. This happens by first mapping the input parameters with a non-linear kernel (here we used Gaussian) and then minimizing the loss that has terms considering both the error to desired output and the number of support vectors (i.e. model complexity). Moreover, the loss is so called ϵ-tube insensitive, i.e. SVM predictions separated by less than ϵ have zero loss. The tube parameter ϵ of the error function for the best fit varied with the different stress-strain values and it was obtained with a grid search.

For both models, all the input data was scaled to have zero mean and unit variance. In total, the number of samples for each set was roughly 5000 (10000 for the systems with 50 initial dislocations). The number of samples varied a bit due to some initial configurations causing the simulation to get stuck. The samples were then divided to training and test sets; in SVM the sets contained 90 and 10 percent of the samples, respectively, while for the ANN, the training set contained 80, validation set 10 and test set 10 percent of the samples.

Supplementary Table 1. **The threshold values of the avalanche size for an avalanche to be examined.** Only the avalanches larger than the threshold are included to the size distributions.

	$N = 50$	$N = 100$	$N = 200$	$N = 300$	$N = 400$
basic	$3.5 \cdot 10^{-4}$	$2.2 \cdot 10^{-4}$	$1.6 \cdot 10^{-4}$	$1.3 \cdot 10^{-4}$	$1.0 \cdot 10^{-4}$
ID	$2.5 \cdot 10^{-4}$	$2.1 \cdot 10^{-4}$	$1.8 \cdot 10^{-4}$	$1.6 \cdot 10^{-4}$	$1.3 \cdot 10^{-4}$

Supplementary Table 2. **Extracted input parameters for the ANN and SVM.** The list of all the used descriptors of the initial dislocation configuration. Parameters f_{xi} refer to Fourier coefficients of ρ_{GND} in x-direction and correspondingly f_{yi} refer to coefficients of ρ_{GND} in y-direction. Naturally, σ_{ID} is used only in the training with the ID systems. The kth Fourier coefficient in for example y-direction is computed by $f_{yk} = \sum_{i=0}^{n} \rho_{\mathrm{GND}}(x,i) \exp\left[-2\pi j \left(\frac{ik}{n+1}\right)\right]$, where $\rho_{\mathrm{GND}}(x,i)$ is the sum of GND density in the ith horizontal slice.

average of σ_{sf}	average of $\lvert\sigma_{\mathrm{sf}}\rvert$
variance of σ_{sf}	variance of $\lvert\sigma_{\mathrm{sf}}\rvert$
skewness of σ_{sf}	median of $\lvert\sigma_{\mathrm{sf}}\rvert$
kurtosis of σ_{sf}	number of dislocations
f_{x1} of ρ_{GND} field	f_{y1} of ρ_{GND} field
f_{x2} of ρ_{GND} field	f_{y2} of ρ_{GND} field
f_{x3} of ρ_{GND} field	f_{y3} of ρ_{GND} field
ρ_{GND} difference in x-direction	ρ_{GND} difference in y-direction
dislocation wall count	maximum wall height
average wall height	stress from ID, σ_{ID}

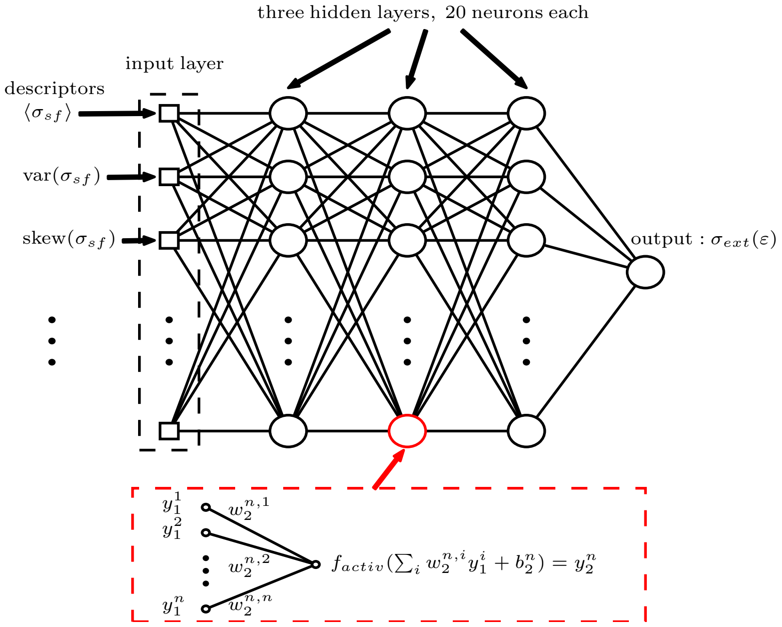

Supplementary Figure 1. **Illustration of the used ANN.** Parameters presented in Supplementary Table 2 are passed to the input layer. The input signal then goes through three fully connected hidden layers, with 20 neurons in each layer, before arriving at the output. To clarify the process, the lower box depicts the passing of the signal through nth neuron in the second hidden layer. Input for the neuron are the values coming from the previous, first hidden layer, and they form the vector $\mathbf{y}_1$. Elements of $\mathbf{y}_1$ are multiplied with the weights of the corresponding connections which form the nth row of matrix $\mathbf{w}_2$ of all connection weights in the second layer. The sum of the products is then added to the bias of the neuron and passed to the activation function. Here, the used activation function is the rectifier, i.e. $f_{activ}(x) = \max(0, x)$. Finally, the activation value is the output of the nth neuron which is passed to every neuron of the subsequent hidden layer.

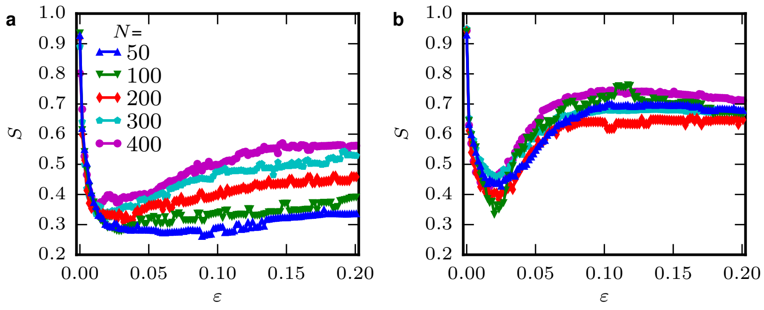

Supplementary Figure 2. **Scores of the SVM fit.** The score of the stress response fit as a function of strain obtained from the SVMs. The general shape of the curves is similar to the output of the ANN: in both **a** the basic and **b** the initially deformed (ID) systems, the score exhibits a local minimum after which the score recovers with larger strains. Additionally the size effect observed in the ANN results appears in SVM fits as well, i.e. the larger systems are more predictable although in the ID case the effect is non-monotonic.

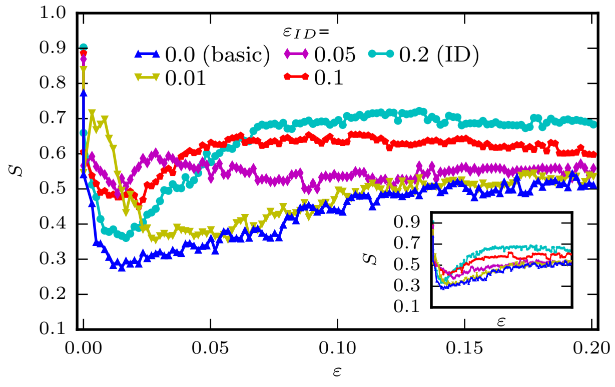

Supplementary Figure 3. **Score of the stress-strain prediction for different pre-strain values.** Number of initial dislocations in the systems is 400. The figure shows that the dislocation systems are more predictable with larger strains when the applied pre-strain is larger, as the initial structures become more correlated during the pre-straining. To show that the addition of σ_{ID} as a descriptor is not entirely responsible for the improved score, the inset illustrates the score from NNs trained without it. Still, the initially deformed are more predictable at larger strains. However, the pre-straining has non-trivial influence on the score during the predictability minimum: the decrease in the score is less dramatic with the smaller, non-zero pre-strain values even without the information about σ_{ID}. This can be thought to arise from the dislocation configurations obtained with certain ε_{ID} having some relevant characteristics only until certain strain, after which the predictability is dictated by the long-range correlations shown in main text. One possibility could be single mobile dislocations which are more common in basic systems and, thus, their effect could be more collective, while in pre-strained systems these could cause relatively large deviations (see also Supplementary Fig. 4).

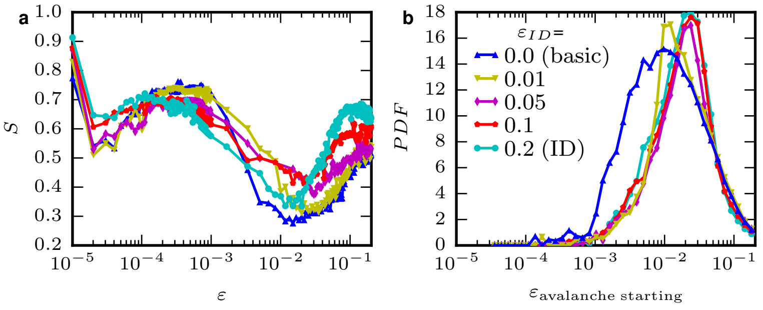

Supplementary Figure 4. **Comparison of the NN scores (without σ_{ID}) and the avalanche starting strain probability distributions for systems with different pre-strain.** The curves show the similarity discussed in the main text: The predictability minima in **a** and distribution maxima in **b** happen with more or less the same strain. Notably, the avalanche activity starts in the basic systems earlier than in any of the pre-strained systems and this is seen in S curves as a steeper decrease. From the ε_{aval} PDFs of the pre-strained systems, only the distribution of $\varepsilon_{ID} = 0.01$ is distinguishable with the only difference being the maximum with smaller strain. Correspondingly around the maximum, the score of $\varepsilon_{ID} = 0.01$ dips lower towards the score of basic systems, after which they are almost equal. This further proves the connection between the predictability and avalanche activity. On the other hand, the score curves with $\varepsilon \sim 10^{-4} \ldots 10^{-3}$ show that here, the less pre-strained systems are momentarily more predictable. The magnitude of these strains is similar to one dislocation moving the average dislocation distance ($\sim 7 \cdot 10^{-4}$) so possibly this is the effect of mobile single dislocations discussed in Supplementary Fig. 3.

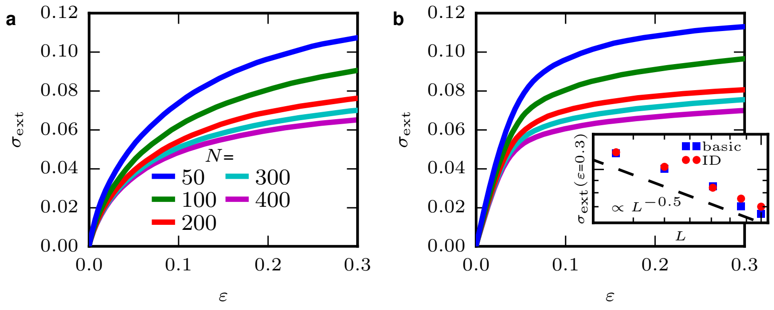

Supplementary Figure 5. **Average stress-strain curves.** The figure shows the average stress response of the approximately 5000 (10000 for $N = 50$) simulated **a** basic and **b** ID systems. The "smaller is stronger" effect is evident; at $\varepsilon = 0.3$, σ_{ext} roughly follows $L^{-0.5}$ where L is the system box size.

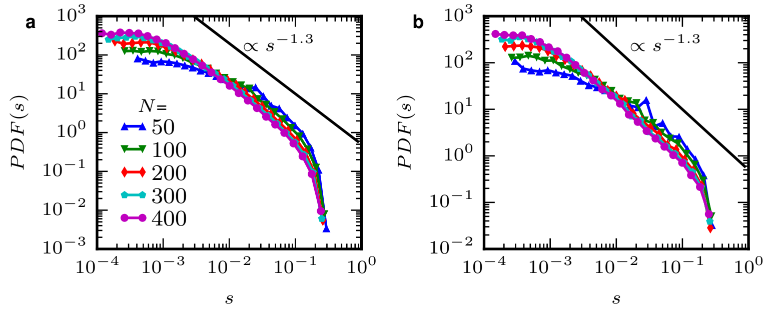

Supplementary Figure 6. **Size distribution of avalanches.** The probability distribution of the avalanche size s in the simulated 2D DDD **a** basic and **b** ID systems. Here only the avalanches, that are distinguishable from the noise caused by the simulation procedure, are examined. The division of the avalanches is illustrated in Supplementary Fig. 10 where the joint distribution of avalanche size and starting strain is plotted. The distributions show no size effect in the cut-off because the systems are simulated to finite strain ($N = 300$ and $N = 400$ to $\varepsilon = 0.3$, other sizes to $\varepsilon = 0.5$) and, thus, the avalanche size is capped at $s = 0.3$.

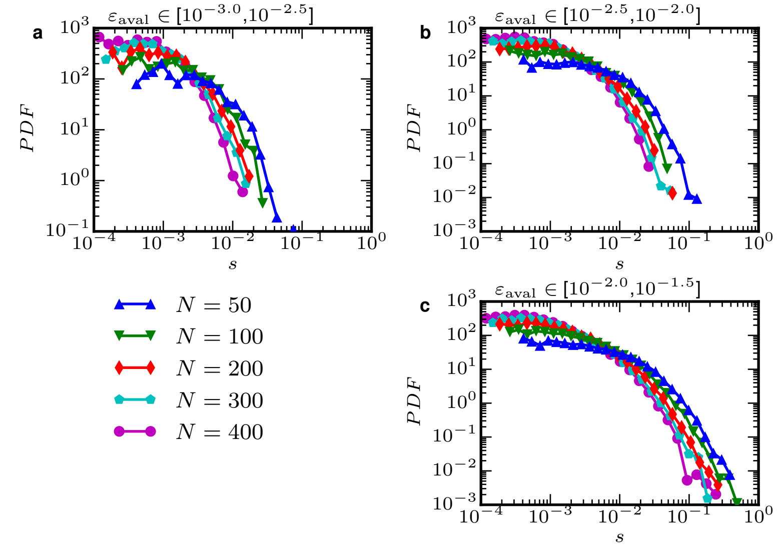

Supplementary Figure 7. **Size distribution of avalanches in basic systems in three strain bins.** The size distribution of all of the avalanches starting in the three strain bins relatively close to the start of the simulation show that as the strain increases, i.e. the simulation proceeds, the distribution cut-off shifts and the systems exhibit larger and larger avalanches. However, a size-effect is evident as in every bin the smaller systems have distributions shifted towards larger s. Here the avalanche size is not capped, as the avalanches start with small strain and, therefore, they are not affected by the finite strain at the end of the simulation as they finish before it.

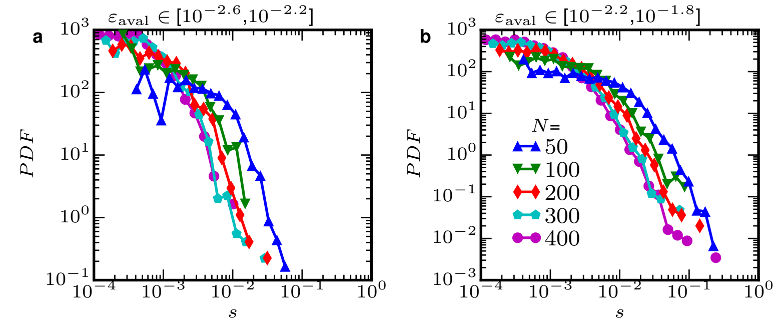

Supplementary Figure 8. **Size distribution of avalanches in ID systems in two strain bins.** The distributions in the two subsequent strain bins have the same features as seen for basic systems in the previous figure: larger avalanches originate later in the simulation, and smaller systems tend to exhibit larger avalanches than the larger systems. Only two strain bins are plotted because the ID systems had fewer avalanches than the basic systems.

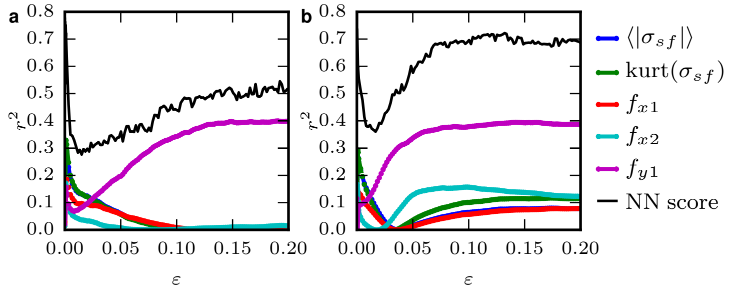

Supplementary Figure 9. **The coefficient of determination r^2, of linear fits between chosen descriptors and the stress σ_{ext} as a function of simulation strain.** Figure **a** contains basic systems and **b** ID systems with 400 initial dislocations. Here, r^2 values are also compared to the score of the NN output (after all, r^2 and score of NN are defined similarly). As seen and discussed in the main text, f_{y1} is by itself the most informative descriptor with large strains. However, it is beaten by the NN score, as other descriptors contain some information, and the used input set possibly contains some latent non-linear dependencies found by the NN.

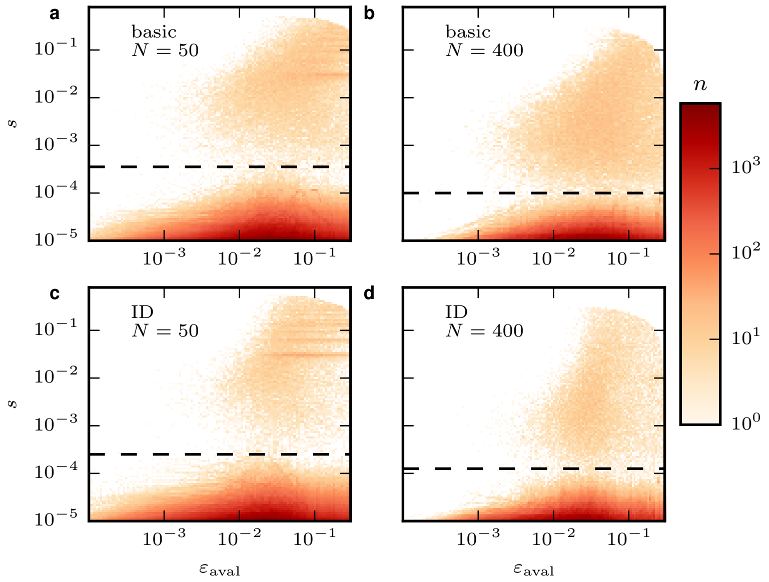

Supplementary Figure 10. **Joint distribution of the avalanche size and the starting strain.** The unnormalized avalanche size and starting strain distribution of **a** basic systems with 50 dislocations, **b** basic systems with 400 dislocations, **c** ID systems with 50 dislocations and **d** ID systems with 400 dislocations all show that the avalanches form two distinct distributions. The numerous small avalanches that start to appear immediately after the beginning of the simulation originate more from the simulation procedure (the dislocation velocity oscillates around the avalanche-defining threshold) than from the bursty collective motion of dislocations so we discarded these from the study. The threshold values varied between sets of systems and they are recorded in Supplementary Table 1, and in the figure they are illustrated by the dashed lines. In the systems of 50 dislocations, the studied avalanche distributions contain peaks that correspond to bursts of one dislocation circulating the simulation box over the periodic boundaries (the smallest size peak is one cycle, the second smallest two cycles etc.). This non-physical behaviour shows that the smallest systems lose validity soon after the start of the stress ramp.

SUPPLEMENTARY REFERENCES

[1] Bishop, C. M. Pattern Recognition and Machine Learning. (Springer, 2006).
[2] Abadi, M. *et al.* TensorFlow: Large-Scale Machine Learning on Heterogeneous Systems. http://tensorflow.org (2015).
[3] Pedregosa, F. *et al.* Scikit-learn: Machine learning in Python. *J. Mach. Learn. Res.* **12**, 2825-2830 (2011).
